# A novel model for accurate and fast prediction of cancer incidence

**DOI:** 10.1186/s12889-025-22624-4

**Published:** 2025-05-06

**Authors:** Mahmoud Hamed, Berlanty A. Zayed, Fotouh R. Mansour

**Affiliations:** 1 Pharmaceutical Chemistry Department, Faculty of Pharmacy, Misr International University, Km 28 Ismailia Road, Cairo, 44971 Egypt; 2 MIU Chemistry Society (MIU-CS), Faculty of Pharmacy, Misr International University, Km 28 Ismailia Road, Cairo, 44971 Egypt; 3 Tanta Student Research Academy, Faculty of Medicine, Tanta University, Tanta, 31111 Egypt; 4 Department of Pharmaceutical Analytical Chemistry, Faculty of Pharmacy, Tanta University, Tanta, 31111 Egypt; 5 Department of Medicinal Chemistry, Faculty of Pharmacy, King Salman International University (KSIU), South Sinai, Egypt

**Keywords:** Prediction model, Cancer incidence, Google Trends, Relative search volume index

## Abstract

**Background:**

Predicting cancer incidence has long been a challenge for clinicians and researchers. Accurate predictions are essential for health planning to ensure adequate resources for diagnosis, treatment, and rehabilitation. Current prediction methods rely on historical data, assuming persistent patterns of cancer incidence.

**Method:**

In this study, the Google Trends tool was used to obtain the relative search volume index (RSVI) for the topic “cancer” each year from 2017 to 2023 in the United States and worldwide. The proposed model incorporated actual cancer incidence rates and yearly changes in RSVI.

**Results:**

The model was applied to predict the rates of new cancer cases in fifty American states over four consecutive years (2017, 2018, 2019, 2020). The selection of years was restricted with data availability. In most states, the percentage error did not exceed 6%. The high degree of similarity between the actual and predicted cancer incidence rates was notable. Similar results were obtained when predicting cancer incidence rates in the countries studied.

**Conclusion:**

The model has successfully provided accurate short-term predictions of cancer incidence rates across all 50 American states and 54 countries since 2017.

**Supplementary Information:**

The online version contains supplementary material available at 10.1186/s12889-025-22624-4.

## Introduction

Cancer is a worldwide leading cause of death, which accounts for about 10 million deaths annually, or about one in every six deaths, according to the World Health Organization (WHO) [1]. The reported cancer incidence rates are 2 to 4 years late behind the current year. The cause for this delay is that data collection, compilation, quality control, and dissemination are not instant processes [2]. Predicting cancer incidence is a crucial step in estimating the cancer burden, which is essential for health planning and ensuring that healthcare organizations allocate sufficient resources for diagnosis, treatment, and rehabilitation [3]. Historically, different methods hava been used for making cancer incidence predictions [4–6]. Age-period-cohort (APC) models have been the most widely used approach in the last four decades to forecast the incidence and the mortality of cancer. In the APC model, the rate of cancer incidence is described as a sum of (non-linear) age, period, and cohort-effects where the period indicates the date of follow-up, and the cohort is indicated by the date of birth. However, these three variables (age, period, and cohort) are linearly co-related according to the equation; Cohort = Period – Age. Accordingly, the effect of any of these variables can be deduced once the other two variables are given. This exact linear dependency among age, period, and cohort leads to a problem known as the “Identification problem”. For this reason, The APC model tends to overestimate the rate of cancer cases. Attempts have been made to overcome this problem, but the model is still complicated and the results are not very reliable [3].

**Fig. 1:**
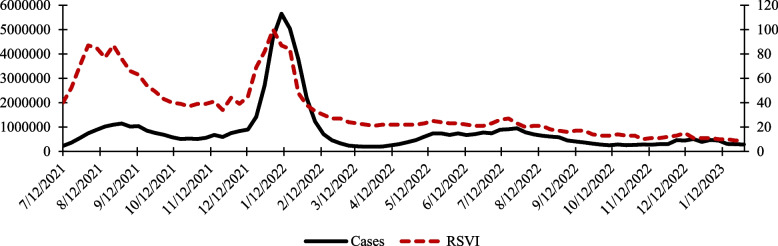
Dual Axis Chart showing the correlation between COVID- 19 cases (Solid black line, left scale) and RSVI using the term “COVID- 19” (Dashed red line, right scale). The rates of cases are accessed from the WHO (covid19.who.int) on 18 August 2023

Many years ago, Google Inc. introduced Google Trends as a brand-new, open-access, cost-free application [7]. The information shown in Google Trends is classified according to the popularity of particular subjects at particular times and locations [8]. The user-friendly interface in Google Trends allows comparing up to five distinct topics at once, as well as the search trends in other nations or areas (Video S1). The results are shown as a chart that illustrates the interest in the query over time [7]. In this graph, the y-axis is the relative search volume (RSV) of the query, and the x-axis represents the time period according to user preference (from 2004 to the present day) [7]. When compared to other subjects that were searched for at the given time and place, RSV shows the relative popularity of the selected topic rather than its absolute popularity [7]. When compared to other subjects searched within that time period, a topic with an RSV score of 100 is at the pinnacle of popularity, while one with a score of 55 indicates that it was only 55% as popular as the most searched for topic at the same time and in the same place [9]. Google Trends also displays regional or country-specific colored maps that demonstrate interest in particular topics on a global scale. Google Trends is widely utilized in a variety of industries, including banking [10], tourism [11], business [12], fashion [13], recreation [14], the oil industry [15], and healthcare [16]. For instance, Google Trends has been used to accurately estimate the direction of stock markets [17], movie engagement [18], fashion consumer behavior [13], sales, and the unemployment rate [19]. Numerous studies in the medical field have explored the potential of using Google Trends to forecast disease outbreaks, including those caused by influenza [20], dengue fever [21], the Middle East respiratory syndrome coronavirus (MERS-CoV) [9], measles [22], Ebola virus [23], and the Zika virus [24]. The link between Google Trends data and how people perceive certain topics can be understood by looking at COVID- 19 cases in the USA according to the WHO (covid19.who.int) and public interest. In Fig. 1, there was a slight increase in the rate of cases from August to September 2021. During this time, more people also showed interest in COVID- 19, likely because of feelings of shock and fear. As time passed, the public's interest (RSVI) became more accurate and sensitive, reflecting real-life situations. This is clear when we look at the big increase in cases in January 2022, which was also matched by a rise in RSVI. Both lines followed a similar pattern, showing a marked correlation between them. In this work, a simple model for short term prediction of cancer incidence rates is presented. The proposed model, named CanTrend, is based on Google Trends data and historical data from health agencies. Compared with the current models for cancer prediction, this proposed model is simpler, faster, and could be more accurate because Google Trends reduces the oversensitivity of linear models to sudden or large changes of observed data, if used alone.

## Methods

### Data sources

The actual rates of new cases (for all the populations worldwide in 2020, for all cancers, both sexes, all ages) were collected from the World Health Organization (WHO, iarc.who.int). The actual data of the United States population-based cancer incidence have been reported by North American Association of Central Cancer Registries (NAACCR) (accessed via CiNA Explorer, August 2023) [25]. The rates of the United States were reported for all ages, all races and for both males and females.

### Method and validation

Google Trends was used to obtain the relative search volume index (RSVI) for the topic; “cancer”. The resultant RSVI indicates the number of searches for a certain topic in a definite time period or in a geographical region relative to the time period or region with the peak search attempts. RSVI is calculated on a scale of 100, where 100 refers to the maximum search frequency, 50 indicates mid-level search frequency, while 0 designates insufficient search data for this topic [26]. The proposed model relies on the actual rates of cancer incidence in NAACCR database and the change in RSVI from year to year. For example, to calculate the predicted rates of cancer incidence in 2017, the actual rates of cancer incidence in NAACCR database in 2016 were multiplied by the ratio RSVI2017/RSVI2016 where RSVI2017 and RSVI2016 are the RSVI values of a selected country or state for the search topic “cancer” in 2017 and 2016, respectively. The accuracy of the predicted rates was assessed by calculating the percentage error according to the equation:

$$\%Error=\frac{\mathit{Pr}-Ac}{Ac}\times 100$$

where Pr is the predicted rate of cases, and Ac is the actual rate of cases in a selected country or state during a pre-defined period according NAACCR database. The model was applied to predict the rates of new cancer cases in fifty U.S. states for four consecutive years (2017, 2018, 2019, 2020). The selection of years was restricted with data availability. The model was applied for predicting the rates of cancer incidence in 50 different American states and in 54 countries.

## Results and discussion

**Fig. 2:**
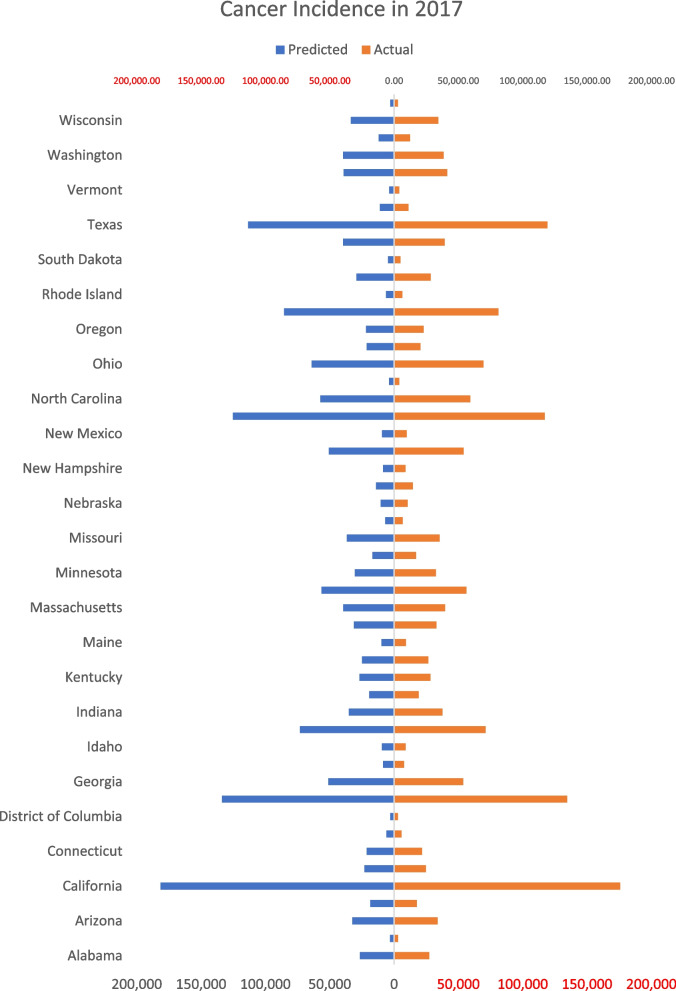
Actual rates for cancer burden in 50 US states in 2017 compared to the predicted rates

The prediction of the cancer burden is crucial for health planning. The nowcasting of cancer incidence helps health care organizations prepare the necessary resources for diagnosis, treatment, and rehabilitation. The proposed model uses the actual data together with the data available on Google Trends to predict cancer incidence. The results in Table 1 show the predicted new cases in 50 US states in 2017 based on the actual data in 2016, and the RSVI in 2016 and 2017. The increase (or decrease) in RSVI is expected to be associated with a corresponding increase (or decrease) in cancer incidence. The %error of prediction was calculated and was found to have a median value of − 0.98%. While some states showed high %error (e.g. Hawaii and Vermont), the %error in most of the states did not exceed 6%. The high level of similarity between the predicted rates for cancer incidence within 2017 can be markedly noticed in Fig. 2, which reflects the reliability of the model in the prediction of new cancer cases. The results of 2018–2020 are shown in Figures S1-S3. To confirm the model validity, the predicted rates for 2018, 2019 and 2020 were calculated and the %errors of predication were compared as shown in Table 2.

**Table 1:** The predicted rates for cancer incidence in 2017 using the actual rates in 2016 the RSVI in 2016 and 2017 via Google Trends

US State	Actual cases in 2016	RSVI_2016_	RSVI_2017_	RSVI_2017_/RSVI_2016_	Predicted Cases for 2017	Actual Cases in 2017	% Error
Alabama	27550	89	86	0.966	26621	27409	− 2.9
Alaska	3008	67	70	1.045	3143	3067	2.5
Arizona	32810	85	84	0.988	32424	33909	− 4.4
Arkansas	17410	80	85	1.063	18498	17722	4.4
California	172652	78	82	1.051	181506	175579	3.4
Colorado	23852	80	77	0.963	22958	24638	− 6.8
Connecticut	21572	95	94	0.989	21345	21731	− 1.8
Delaware	6101	88	85	0.966	5893	5804	1.5
District of Columbia	2745	78	83	1.064	2921	2966	− 1.5
Florida	135354	82	81	0.988	133703	134514	− 0.6
Georgia	52992	87	84	0.966	51165	53729	− 4.8
Hawaii	7502	68	78	1.147	8605	7677	12.1
Idaho	8670	75	82	1.093	9479	8996	5.4
Illinois	70577	81	84	1.037	73191	71140	2.9
Indiana	36765	90	86	0.956	35131	37614	− 6.6
Iowa	18850	81	83	1.025	19315	19188	0.7
Kentucky	27720	95	92	0.968	26845	28219	− 4.9
Louisiana	25878	82	79	0.963	24931	26477	− 5.8
Maine	9061	87	94	1.080	9790	9228	6.1
Maryland	31916	92	90	0.978	31222	32949	− 5.2
Massachusetts	39427	90	90	1.000	39427	39623	− 0.5
Michigan	56973	86	85	0.988	56311	56181	0.2
Minnesota	30505	84	84	1.000	30505	32369	− 5.8
Mississippi	16833	86	86	1.000	16833	17067	− 1.4
Missouri	33869	86	93	1.081	36626	35359	3.6
Montana	6430	73	78	1.068	6870	6647	3.4
Nebraska	10337	86	87	1.012	10457	10578	− 1.1
Nevada	13616	77	79	1.026	13970	14621	− 4.5
New Hampshire	8692	88	86	0.977	8494	8774	− 3.2
New Jersey	52962	91	87	0.956	50634	54033	− 6.3
New Mexico	9700	81	78	0.963	9341	9745	− 4.1
New York	115030	90	98	1.089	125255	117153	6.9
North Carolina	57398	83	83	1.000	57398	59104	− 2.9
North Dakota	3871	77	78	1.013	3921	3987	− 1.6
Ohio	67791	91	86	0.945	64066	69367	− 7.6
Oklahoma	20499	81	84	1.037	21258	20431	4.0
Oregon	22118	77	76	0.987	21831	23004	− 5.1
Pennsylvania	81244	94	99	1.053	85565	81018	5.6
Rhode Island	6343	87	85	0.977	6197	6442	− 3.8
South Carolina	28230	85	88	1.035	29226	28439	2.8
South Dakota	4811	90	88	0.978	4704	4865	− 3.3
Tennessee	37864	86	90	1.047	39625	39350	0.7
Texas	114969	76	75	0.987	113456	119174	− 4.8
Utah	10724	73	75	1.027	11018	11168	− 1.3
Vermont	3829	83	81	0.976	3737	4034	− 7.4
Virginia	41108	85	81	0.953	39174	41366	− 5.3
Washington	38132	72	75	1.042	39721	38448	3.3
West Virginia	12032	100	100	1.000	12032	12492	− 3.7
Wisconsin	33979	85	84	0.988	33579	34305	− 2.1
Wyoming	2802	68	72	1.059	2967	2891	2.6

**Table 2:** The predicted cancer incidence in 2018–2020 using the developed model

US State	2018			2019			2020		
	**Predicted**	**Actual**	**% Error**	**Predicted**	**Actual**	**% Error**	**Predicted**	**Actual**	**% Error**
**Alabama**	30915	27463	12.6	26331	27971	− 5.9	26166	25348	3.2
**Alaska**	3067	3128	− 2.0	3128	3307	− 5.4	3165	3110	1.8
**Arizona**	34313	34797	− 1.4	35206	35159	0.1	33524	32171	4.2
**Arkansas**	18139	18505	− 2.0	16378	18701	− 12.4	19187	15482	23.9
**California**	162732	176082	− 7.6	176082	182033	− 3.3	179638	163739	9.7
**Colorado**	23038	25135	− 8.3	25484	26180	− 2.7	26897	24212	11.1
**Connecticut**	21269	21471	− 0.9	21004	21712	− 3.3	20747	19861	4.5
**Delaware**	5941	6067	− 2.1	6206	6306	− 1.6	5810	5535	5.0
**District of Columbia**	2823	2907	− 2.9	2907	2905	0.1	2648	2585	2.4
**Florida**	144478	140026	3.2	138417	139607	− 0.9	133114	130258	2.2
**Georgia**	53729	55038	− 2.4	57659	56527	2.0	51388	53486	− 3.9
**Hawaii**	7283	7651	− 4.8	7754	7819	− 0.8	7923	7285	8.8
**Idaho**	8557	9088	− 5.8	8855	9648	− 8.2	9394	9208	2.0
**Illinois**	73681	71351	3.3	68891	72336	− 4.8	68030	65398	4.0
**Indiana**	39363	37021	6.3	37021	35833	3.3	33046	28075	17.7
**Iowa**	18494	19443	− 4.9	20172	20125	0.2	18913	18381	2.9
**Kentucky**	26685	28475	− 6.3	29784	28899	3.1	27629	26240	5.3
**Louisiana**	27818	27404	1.5	26413	28003	− 5.7	26253	24693	6.3
**Maine**	9228	9282	− 0.6	9381	9741	− 3.7	8511	9151	− 7.0
**Maryland**	31119	33335	− 6.6	33727	34998	− 3.6	34591	30603	13.0
**Massachusetts**	38742	38408	0.9	40154	40842	− 1.7	37291	34966	6.6
**Michigan**	58825	57003	3.2	54441	57490	− 5.3	53432	51987	2.8
**Minnesota**	30057	32812	− 8.4	35336	33600	5.2	34400	30507	12.8
**Mississippi**	18456	17407	6.0	17033	17456	− 2.4	14387	15145	− 5.0
**Missouri**	33078	34772	− 4.9	37170	35235	5.5	32583	33694	− 3.3
**Montana**	6817	6624	2.9	6541	6560	− 0.3	5896	6045	− 2.5
**Nebraska**	10456	10902	− 4.1	10775	10773	0.0	10773	9281	16.1
**Nevada**	14621	13630	7.3	13630	13077	4.2	13077	14149	− 7.6
**New Hampshire**	8876	8928	− 0.6	9133	9266	− 1.4	8537	8177	4.4
**New Jersey**	52791	54430	− 3.0	53149	55269	− 3.8	54603	50347	8.5
**New Mexico**	9245	9740	− 5.1	10793	10143	6.4	9525	9031	5.5
**New York**	117153	116333	0.7	118707	120228	− 1.3	120228	105600	13.9
**North Carolina**	58392	60057	− 2.8	62987	62277	1.1	60829	57243	6.3
**North Dakota**	4038	3934	2.6	3934	4024	− 2.2	3973	3843	3.4
**Ohio**	70174	70851	− 1.0	74109	71897	3.1	62416	65151	− 4.2
**Oklahoma**	20188	21037	− 4.0	21797	21527	1.3	20776	19782	5.0
**Oregon**	24517	22632	8.3	21794	23745	− 8.2	21614	20141	7.3
**Pennsylvania**	73653	80903	− 9.0	83600	82588	1.2	77260	72403	6.7
**Rhode Island**	6669	6561	1.7	6561	6747	− 2.8	6210	5552	11.9
**South Carolina**	27469	28610	− 4.0	30630	28875	6.1	25385	27362	− 7.2
**South Dakota**	4533	4958	− 8.6	4898	5150	− 4.9	4451	4738	− 6.1
**Tennessee**	38476	38993	− 1.3	43424	39287	10.5	33675	36396	− 7.5
**Texas**	122352	124867	− 2.0	128110	127456	0.5	125843	116875	7.7
**Utah**	10572	11748	− 10	11913	12002	− 0.7	11502	11602	− 0.9
**Vermont**	3934	4039	− 2.6	3937	4073	− 3.3	3967	3710	6.9
**Virginia**	42387	41537	2.0	42037	43797	− 4.0	42754	39599	8.0
**Washington**	37423	39120	− 4.3	42871	39859	7.6	35873	36018	− 0.4
**West Virginia**	12492	12539	− 0.4	12539	12362	1.4	11249	11445	− 1.7
**Wisconsin**	33488	34615	− 3.3	39258	36078	8.8	30647	32962	− 7.0
**Wyoming**	2690	2858	− 5.9	2986	3051	− 2.1	2789	2857	− 2.4

**Fig. 3:**
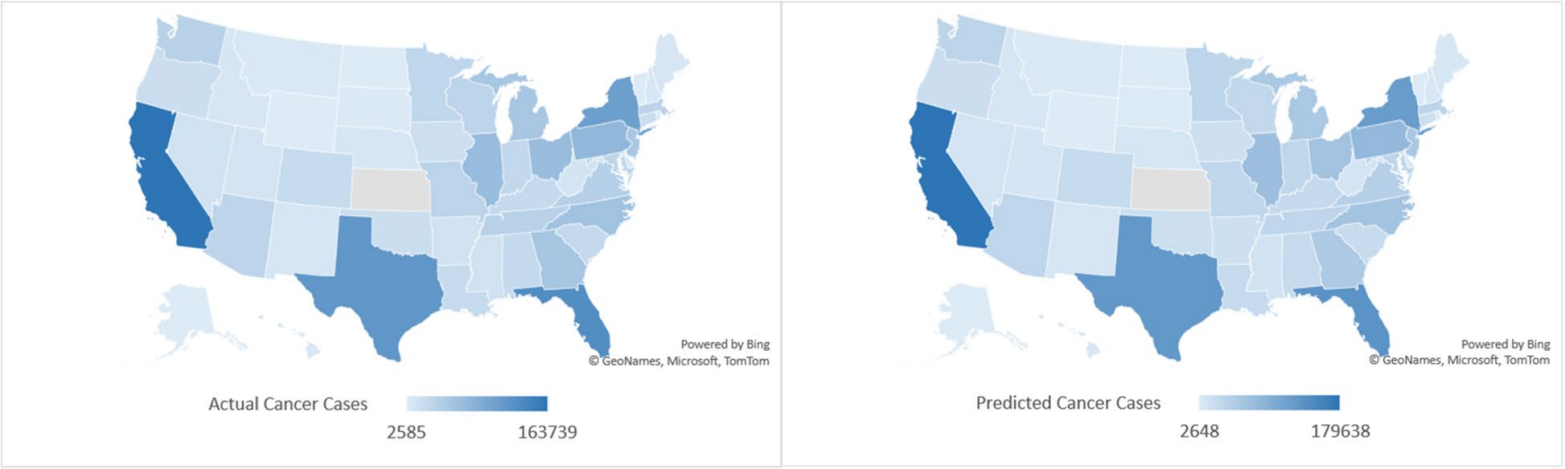
The US heat map showing the actual (left) and the predicted new cancer cases (right) in 2020

The following figure shows the US heat map for the actual and the predicted cancer cases in 2020. The high similarity between the actual rates of cases and the predicted rates shown in Fig. 3 indicates the reliability of the developed model. Similar results were obtained for the years 2017–2019 as shown in Figures S4-S6.

**Fig. 4:**
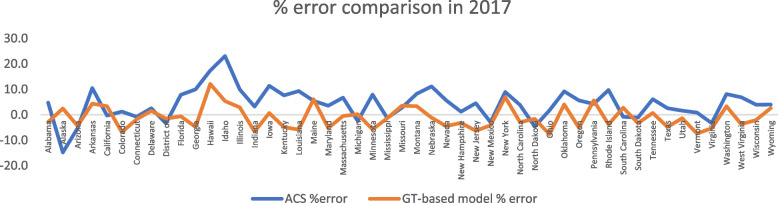
A comparison between the % error of our proposed Google Trends (GT)-based model and the model used by Siegel et al., published by the American Cancer Society (ACS) in 2017

To facilitate a comparative analysis between our Google Trends-based model and the approach adopted by the American Cancer Society [27–30], a line graphs depicting the %errors of both models has been included to cover the time span in 2017 in Fig. 4 and from 2018 to 2020 in Figure S7-S9. The concept behind this cancer prediction model involves utilizing population-based data on cancer incidence and mortality in the United States to estimate and analyze new cancer cases and deaths. By incorporating epidemiological principles and statistical methods, the model can track trends in cancer rates over time.

**Fig. 5:**
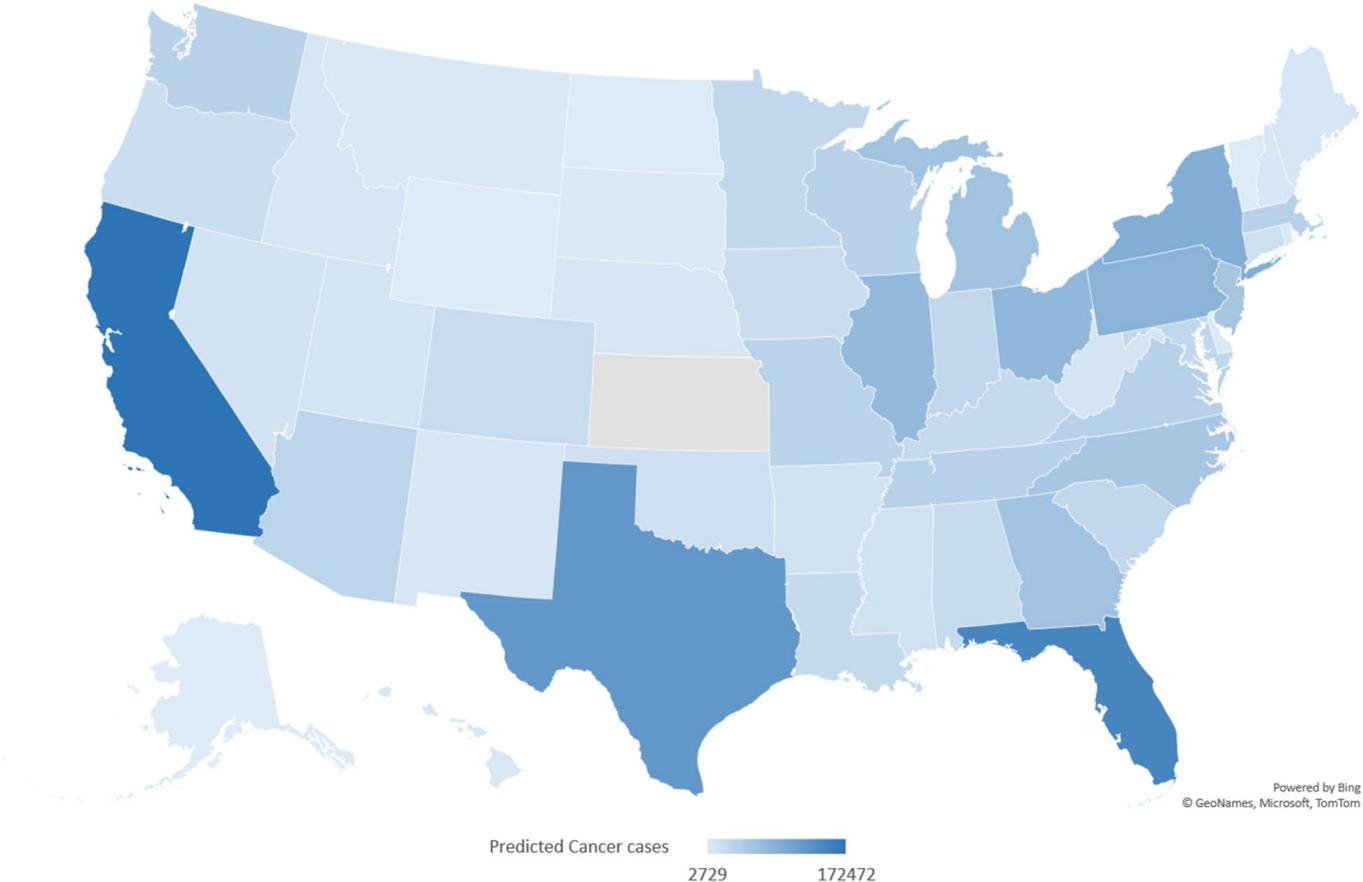
US States map showing the states with the highest rates of cancer cases expressed by dark blue and states with lowest rates expressed by light blue in 2023 (till August)

Given the COVID- 19 pandemic's onset in 2019, health services have encountered partial or complete disruptions in various nations. Such disruptions could potentially account for the delay in capturing cancer incidence data since 2019 via the WHO website. This highlights the significance of our model in addressing challenges posed by such circumstances. Fear and concern were the motives to switch the interest from cancer to COVID- 19. To overcome this problem, we used the last predicted data available to project the expected rates of cancer cases in the next year. For example, the actual case rates for 2021 were not available on the WHO website at the time of submitting this study. Our model could predict cancer incidence in 2021, based on actual data in 2020, and the RSVIs in 2020 and 2021. To predict cancer incidence in 2021, the expected rates in 2021 were employed together with the RSVIs in 2021 and 2022. The Same principle was applied to predict cancer incidence in 2023 in the American states, as shown in Fig. 5. Although this approach overcomes the problem of data unavailability, The accuracy cannot be assessed as the actual data were not available at the time of submitting this study. Moreover, the estimated %error is expected to increase due to the cumulative deviation from reality from year to year. This problem can be solved once the actual data for the last year are accessible. Table 3 shows the predicted rates of new cancer cases for the fifty states in 2021, 2022, and 2023.

**Table 3:** The Predicted new cancer cases for the fifty states in 2021–2023

US State	2021	2022	2023
Alabama	28553	28553	26222
Alaska	3017	3435	2971
Arizona	30994	34133	34133
Arkansas	16658	17246	16462
California	170289	172472	172472
Colorado	23244	22921	23889
Connecticut	20092	21016	18706
Delaware	5805	6143	5873
District of Columbia	2585	2765	2729
Florida	135024	139789	147732
Georgia	52817	54155	60172
Hawaii	6902	7285	7285
Idaho	9457	9955	10577
Illinois	68709	67881	73676
Indiana	29766	29428	30781
Iowa	18617	20266	22387
Kentucky	26240	26843	27145
Louisiana	25351	29302	27656
Maine	10143	9813	10364
Maryland	30603	30963	31323
Massachusetts	38712	37464	39545
Michigan	53961	56593	60542
Minnesota	30152	29088	30507
Mississippi	16760	17972	16962
Missouri	33694	35261	36437
Montana	5790	6215	6896
Nebraska	9390	9718	9718
Nevada	13791	15045	14686
New Hampshire	7978	8676	8177
New Jersey	51575	50961	56487
New Mexico	9031	9266	9852
New York	105600	91872	90816
North Carolina	54517	57924	55199
North Dakota	3498	3843	3942
Ohio	71749	74223	76697
Oklahoma	18590	20020	19544
Oregon	21559	21559	21843
Pennsylvania	77396	77396	83222
Rhode Island	5483	6169	6237
South Carolina	26678	30440	31466
South Dakota	5550	5686	5483
Tennessee	35529	38562	38562
Texas	112380	124367	121370
Utah	11938	11938	13115
Vermont	4155	4106	4155
Virginia	37184	40082	39599
Washington	38019	37019	38519
West Virginia	12577	12577	12577
Wisconsin	36300	35465	37969
Wyoming	2991	3080	2946

**Fig. 6:**
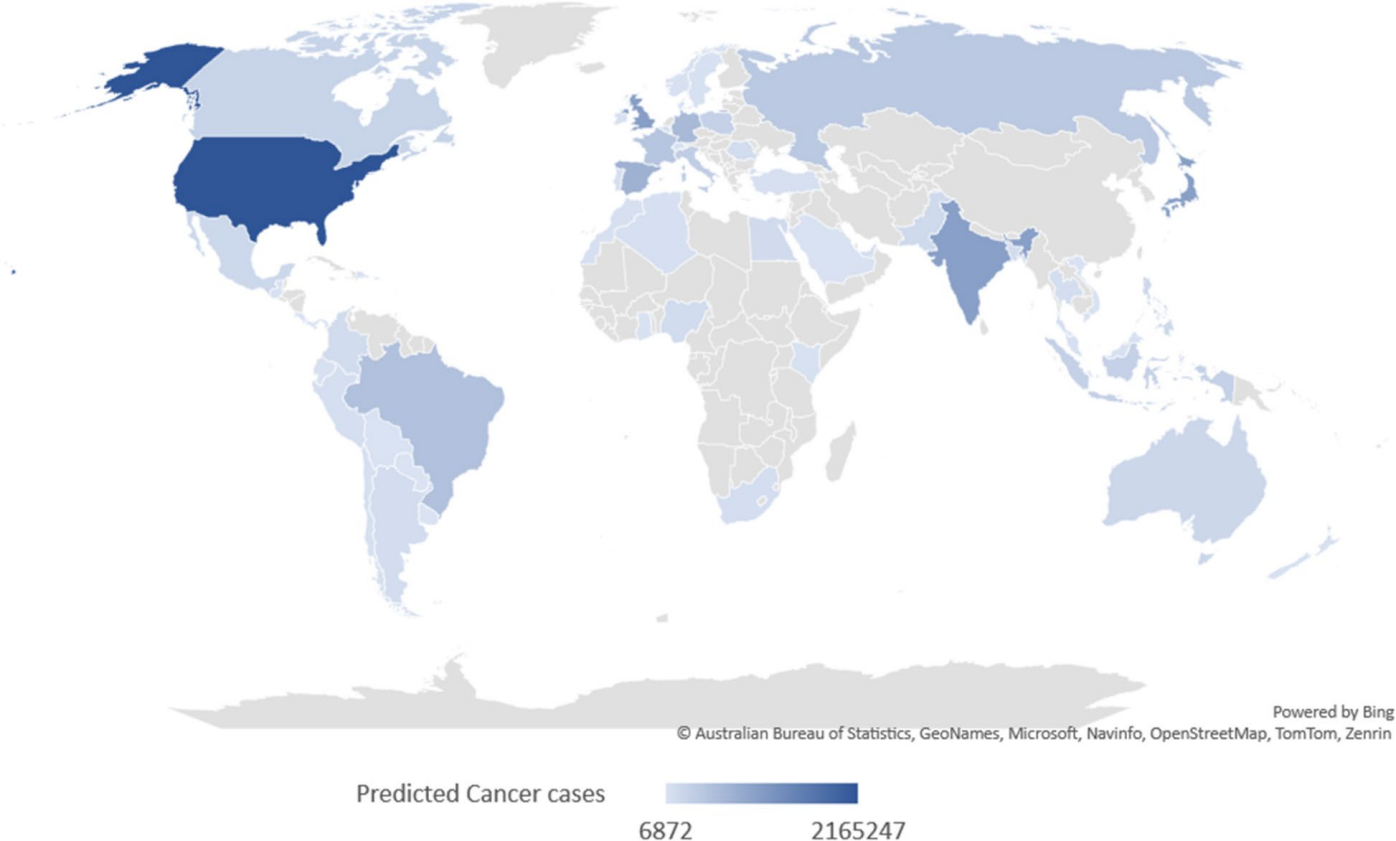
World map showing countries with the highest rates of cancer cases expressed by dark blue and countries with lowest rates expressed by light blue in 2023 (till August)

The same protocol is applied to 54 different populations in different countries to predict new cancer cases in 2021. The predicted new cancer cases are shown in Table 4. According to our findings, the United States of America has the highest cancer burden in 2023 with 2,165,247 new cases followed by the United Kingdom in the second place with 1,040,818 new cases (Fig. 6).

**Table 4:** The Predicted rates of new cancer cases in 2021–2023 worldwide

Country	Predicted cancer cases in 2021	Predicted cancer cases in 2022	Predicted cancer cases in 2023
Algeria	56081	58418	51408
Argentina	109065	99370	92099
Australia	184635	197823	184635
Bangladesh	137962	137962	112878
Belgium	80293	86241	80293
Bolivia	13404	15549	14745
Brazil	485614	509302	521147
Canada	255657	252540	240069
Chile	49470	53276	53276
Colombia	101176	105994	115630
Costa Rica	11467	12661	12422
Denmark	36457	40746	34313
Dominican Republic	16865	18130	16021
Ecuador	28622	32526	29273
Egypt	113919	134632	124276
France	426915	459755	443335
Germany	502815	502815	628519
Ghana	17340	21675	19674
Guatemala	14930	16247	15808
India	1023410	1173912	1023410
Indonesia	272878	272878	272878
Ireland	32187	29200	28869
Italy	332215	332215	332215
Japan	1028658	2057316	1028658
Kenya	34573	43373	42745
Malaysia	34559	37119	38399
Mexico	177726	188390	177726
Morocco	54207	56789	49045
New Zealand	33769	30739	30306
Nigeria	102121	131623	118007
Norway	32721	37083	34902
Pakistan	158567	162531	146675
Panama	7007	7816	6872
Paraguay	10571	11745	10571
Peru	62088	69849	71143
Philippines	129727	148946	163360
Poland	136383	204575	272767
Portugal	57108	60467	60467
Puerto Rico	12434	12757	12757
Romania	96061	98886	104537
Russian Federation	394247	394247	394247
Saudi Arabia	23595	25740	23595
Singapore	21302	20304	21968
South Africa	86221	90924	84653
Spain	815883	784503	753123
Sweden	40566	38374	39470
Switzerland	30242	33266	30242
Thailand	86653	103983	86653
Turkey	46767	70150	46767
United Arab Emirates	59286	62491	59286
United Kingdom	1040818	1040818	1040818
United States of America	2048836	2211811	2165247
Uruguay	8036	8351	6933
Viet Nam	18629	14903	14903

## Conclusion

A novel model for cancer incidence prediction was developed using data from Google Trends. The model could successfully make accurate short-term prediction of the cancer incidence rates in 50 American states and in 54 different countries since 2017. The results were compared with actual incidence rates, and the % relative errors were calculated. The model showed high accuracy, simplicity, and reliability. These findings could be helpful for health care teams to set plans for diagnosis and treatment of cancer. However, this model was not used for long term prediction because it depends on the availability of data from searches on Google Trends. Yet, the model saves time, and effort plus being helpful to overcome the problem of the time lag in providing actual incidence rates of cancer.

## Supplementary Information

Supplementary Material 1.

Supplementary Material 2.

Supplementary Material 3.

## Data Availability

The datasets generated and analyzed during the current study are available in the manuscript and in the supplementary material. All data are available upon reasonable request.
