# Supplementary material for: A novel model for accurate and fast prediction of cancer incidence
Source: BMC Public Health. 2025 May 6;25:1671. doi: 10.1186/s12889-025-22624-4 (PMC12053845; doi:10.1186/s12889-025-22624-4)
Supplement: Supplementary file 2 — Supplementary Material 2. [file 12889_2025_22624_MOESM2_ESM.docx]

**A novel model for accurate and fast prediction of cancer incidence**

Mahmoud Hamed^1,2^, Berlanty A. Zayed^3^, Fotouh R. Mansour^4,5*^

*^1^Pharmaceutical Chemistry Department, Faculty of Pharmacy, Misr International University, Km 28 Ismailia Road, Cairo, 44971, Egypt*

*^2^MIU Chemistry Society (MIU-CS), Faculty of Pharmacy, Misr International University, Km 28 Ismailia Road, Cairo, 44971, Egypt*

*^3^Tanta Student Research Academy, Faculty of Medicine, Tanta University, Tanta, Egypt, 31111*

*^4^Department of Medicinal Chemistry, Faculty of Pharmacy, King Salman International University (KSIU), South Sinai, Egypt*

*^5^Department of Pharmaceutical Analytical Chemistry, Faculty of Pharmacy, Tanta University, Tanta, Egypt, 31111*

**Supplementary materials**

**Fig. S1:** Actual numbers for cancer burden in 50 US states in 2018 compared with the Predicted numbers.

**Fig. S2:** Actual numbers for cancer burden in 50 US states in 2019 compared with the predicted numbers.

**Fig. S3:** Actual numbers for cancer burden in 50 US states in 2020 compared with the Predicted numbers.


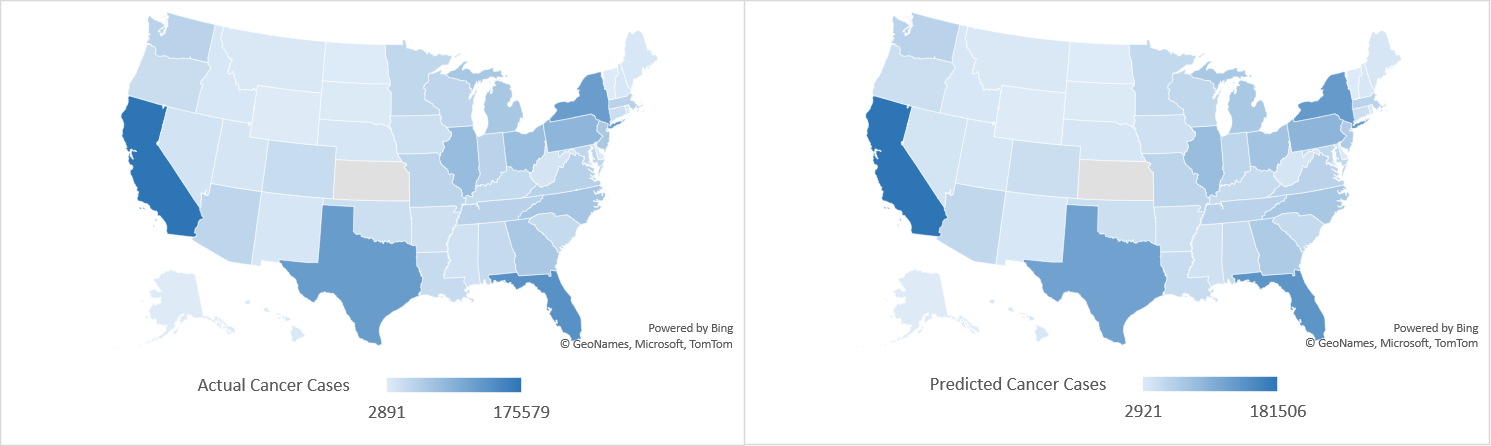


**Fig. S4:** The US heat map showing the actual (left) and the predicted new cancer cases (right) in 2017.


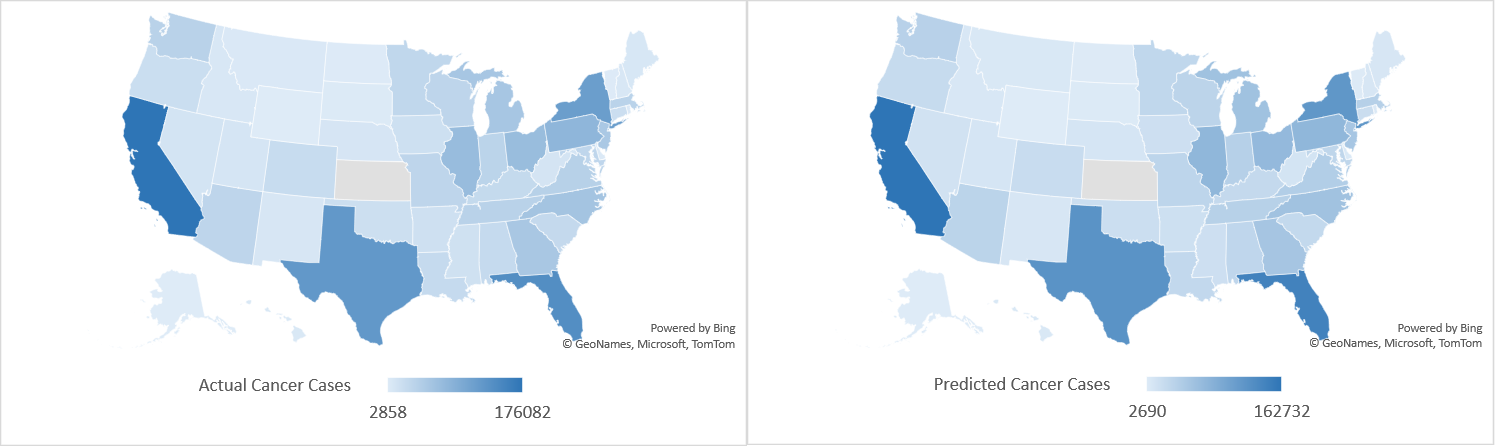


**Fig. S5:** The US heat map showing the actual (left) and the predicted new cancer cases (right) in 2018.


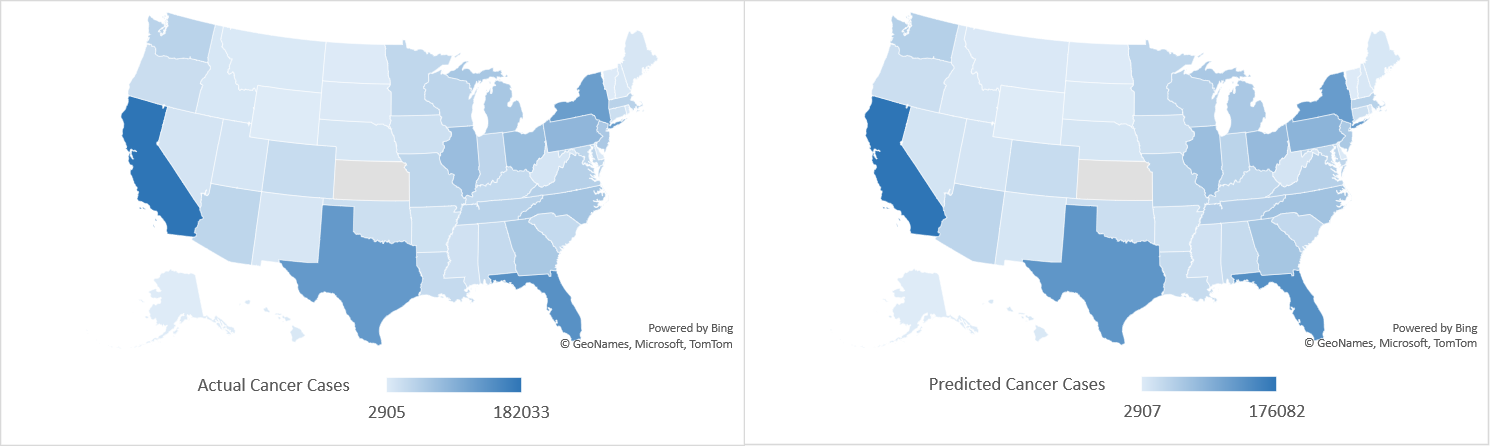


**Fig. S6:** The US heat map showing the actual (left) and the predicted new cancer cases (right) in 2019.

**Fig. S7:** A comparison between the % error of our proposed Google Trends (GT)-based model and the model used by Siegel et al, published by the American Cancer Society (ACS) in 2018.

**Fig. S8:** A comparison between the % error of our proposed Google Trends (GT)-based model and the model used by Siegel et al, published by the American Cancer Society (ACS) in 2019.

**Fig. S9:** A comparison between the % error of our proposed Google Trends (GT)-based model and the model used by Siegel et al, published by the American Cancer Society (ACS) in 2020.
