# Supplementary material for: A novel model for accurate and fast prediction of cancer incidence
Source: BMC Public Health. 2025 May 6;25:1671. doi: 10.1186/s12889-025-22624-4 (PMC12053845; doi:10.1186/s12889-025-22624-4)
Supplement: Supplementary file 3 — Supplementary Material 3. [file 12889_2025_22624_MOESM3_ESM.pdf]

US state	2016		2017					2018					2019					
	RSDI I (2016)	Actual	RSDI II (2017)	RSDI II/RSDI I	Predicted	Actual	% Error	RSDI II (2018)	RSDI II/RSDI I	Predicted	Actual	% Error	RSDI II (2019)	SDI II/RSDI	Predicted	Actual	% Error	RSDI II (2020)
Alabama	89	27550	86	0.966	26621	27409	-2.9	97	1.128	30915	27463	12.6	93	0.959	26331	27971	-5.9	87
Alaska	67	3008	70	1.045	3143	3067	2.5	70	1.000	3067	3128	-2.0	70	1.000	3128	3307	-5.4	67
Arizona	85	32810	84	0.988	32424	33909	-4.4	85	1.012	34313	34797	-1.4	86	1.012	35206	35159	0.1	82
Arkansas	80	17410	85	1.063	18498	17722	4.4	87	1.024	18139	18505	-2.0	77	0.885	16378	18701	-12.4	79
California	78	172652	82	1.051	181506	175579	3.4	76	0.927	162732	176082	-7.6	76	1.000	176082	182033	-3.3	75
Colorado	80	23852	77	0.963	22958	24638	-6.8	72	0.935	23038	25135	-8.3	73	1.014	25484	26180	-2.7	75
Connecticut	95	21572	94	0.989	21345	21731	-1.8	92	0.979	21269	21471	-0.9	90	0.978	21004	21712	-3.3	86
Delaware	88	6101	85	0.966	5893	5804	1.5	87	1.024	5941	6067	-2.1	89	1.023	6206	6306	-1.6	82
District of Columbia	78	2745	83	1.064	2921	2966	-1.5	79	0.952	2823	2907	-2.9	79	1.000	2907	2905	0.1	72
Florida	82	135354	81	0.988	133703	134514	-0.6	87	1.074	144478	140026	3.2	86	0.989	138417	139607	-0.9	82
Georgia	87	52992	84	0.966	51165	53729	-4.8	84	1.000	53729	55038	-2.4	88	1.048	57659	56527	2.0	80
Hawaii	68	7502	78	1.147	8605	7677	12.1	74	0.949	7283	7651	-4.8	75	1.014	7754	7819	-0.8	76
Idaho	75	8670	82	1.093	9479	8996	5.4	78	0.951	8557	9088	-5.8	76	0.974	8855	9648	-8.2	74
Illinois	81	70577	84	1.037	73191	71140	2.9	87	1.036	73681	71351	3.3	84	0.966	68891	72336	-4.8	79
Indiana	90	36765	86	0.956	35131	37614	-6.6	90	1.047	39363	37021	6.3	90	1.000	37021	35833	3.3	83
Iowa	81	18850	83	1.025	19315	19188	0.7	80	0.964	18494	19443	-4.9	83	1.038	20172	20125	0.2	78
Kentucky	95	27720	92	0.968	26845	28219	-4.9	87	0.946	26685	28475	-6.3	91	1.046	29784	28899	3.1	87
Louisiana	82	25878	79	0.963	24931	26477	-5.8	83	1.051	27818	27404	1.5	80	0.964	26413	28003	-5.7	75
Maine	87	9061	94	1.080	9790	9228	6.1	94	1.000	9228	9282	-0.6	95	1.011	9381	9741	-3.7	83
Maryland	92	31916	90	0.978	31222	32949	-5.2	85	0.944	31119	33335	-6.6	86	1.012	33727	34998	-3.6	85
Massachusetts	90	39427	90	1.000	39427	39623	-0.5	88	0.978	38742	38408	0.9	92	1.045	40154	40842	-1.7	84
Michigan	86	56973	85	0.988	56311	56181	0.2	89	1.047	58825	57003	3.2	85	0.955	54441	57490	-5.3	79
Minnesota	84	30505	84	1.000	30505	32369	-5.8	78	0.929	30057	32812	-8.4	84	1.077	35336	33600	5.2	86
Mississippi	86	16833	86	1.000	16833	17067	-1.4	93	1.081	18456	17407	6.0	91	0.978	17033	17456	-2.4	75
Missouri	86	33869	93	1.081	36626	35359	3.6	87	0.935	33078	34772	-4.9	93	1.069	37170	35235	5.5	86
Montana	73	6430	78	1.068	6870	6647	3.4	80	1.026	6817	6624	2.9	79	0.988	6541	6560	-0.3	71
Nebraska	86	10337	87	1.012	10457	10578	-1.1	86	0.989	10456	10902	-4.1	85	0.988	10775	10773	0.0	85
Nevada	77	13616	79	1.026	13970	14621	-4.5	79	1.000	14621	13630	7.3	79	1.000	13630	13077	4.2	79
New Hampshire	88	8692	86	0.977	8494	8774	-3.2	87	1.012	8876	8928	-0.6	89	1.023	9133	9266	-1.4	82
New Jersey	91	52962	87	0.956	50634	54033	-6.3	85	0.977	52791	54430	-3.0	83	0.976	53149	55269	-3.8	82
New Mexico	81	9700	78	0.963	9341	9745	-4.1	74	0.949	9245	9740	-5.1	82	1.108	10793	10143	6.4	77
New York	90	115030	98	1.089	125255	117153	6.9	98	1.000	117153	116333	0.7	100	1.020	118707	120228	-1.3	100
North Carolina	83	57398	83	1.000	57398	59104	-2.9	82	0.988	58392	60057	-2.8	86	1.049	62987	62277	1.1	84
North Dakota	77	3871	78	1.013	3921	3987	-1.6	79	1.013	4038	3934	2.6	79	1.000	3934	4024	-2.2	78
Ohio	91	67791	86	0.945	64066	69367	-7.6	87	1.012	70174	70851	-1.0	91	1.046	74109	71897	3.1	79
Oklahoma	81	20499	84	1.037	21258	20431	4.0	83	0.988	20188	21037	-4.0	86	1.036	21797	21527	1.3	83
Oregon	77	22118	76	0.987	21831	23004	-5.1	81	1.066	24517	22632	8.3	78	0.963	21794	23745	-8.2	71
Pennsylvania	94	81244	99	1.053	85565	81018	5.6	90	0.909	73653	80903	-9.0	93	1.033	83600	82588	1.2	87
Rhode Island	87	6343	85	0.977	6197	6442	-3.8	88	1.035	6669	6561	1.7	88	1.000	6561	6747	-2.8	81
South Carolina	85	28230	88	1.035	29226	28439	2.8	85	0.966	27469	28610	-4.0	91	1.071	30630	28875	6.1	80
South Dakota	90	4811	88	0.978	4704	4865	-3.3	82	0.932	4533	4958	-8.6	81	0.988	4898	5150	-4.9	70
Tennessee	86	37864	90	1.047	39625	39350	0.7	88	0.978	38476	38993	-1.3	98	1.114	43424	39287	10.5	84
Texas	76	114969	75	0.987	113456	119174	-4.8	77	1.027	122352	124867	-2.0	79	1.026	128110	127456	0.5	78
Utah	73	10724	75	1.027	11018	11168	-1.3	71	0.947	10572	11748	-10.0	72	1.014	11913	12002	-0.7	69
Vermont	83	3829	81	0.976	3737	4034	-7.4	79	0.975	3934	4039	-2.6	77	0.975	3937	4073	-3.3	75
Virginia	85	41108	81	0.953	39174	41366	-5.3	83	1.025	42387	41537	2.0	84	1.012	42037	43797	-4.0	82
Washington	72	38132	75	1.042	39721	38448	3.3	73	0.973	37423	39120	-4.3	80	1.096	42871	39859	7.6	72
West Virginia	100	12032	100	1.000	12032	12492	-3.7	100	1.000	12492	12539	-0.4	100	1.000	12539	12362	1.4	91
Wisconsin	85	33979	84	0.988	33579	34305	-2.1	82	0.976	33488	34615	-3.3	93	1.134	39258	36078	8.8	79
Wyoming	68	2802	72	1.059	2967	2891	2.6	67	0.931	2690	2858	-5.9	70	1.045	2986	3051	-2.1	64

2020				2021			2022			2023		
SDI II/RSDI	Predicted	Actual	% Error	RSDI II (2021)	SDI II/RSDI	Predicted	RSDI II (2022)	SDI II/RSDI	Predicted	RSDI II (2023)	SDI II/RSDI	Predicted
0.935	26166	25348	3.2	98	1.126	28553	98	1.000	28553	90	0.918	26222
0.957	3165	3110	1.8	65	0.970	3017	74	1.138	3435	64	0.865	2971
0.953	33524	32171	4.2	79	0.963	30994	87	1.101	34133	87	1.000	34133
1.026	19187	15482	23.9	85	1.076	16658	88	1.035	17246	84	0.955	16462
0.987	179638	163739	9.7	78	1.040	170289	79	1.013	172472	79	1.000	172472
1.027	26897	24212	11.1	72	0.960	23244	71	0.986	22921	74	1.042	23889
0.956	20747	19861	4.5	87	1.012	20092	91	1.046	21016	81	0.890	18706
0.921	5810	5535	5.0	86	1.049	5805	91	1.058	6143	87	0.956	5873
0.911	2648	2585	2.4	72	1.000	2585	77	1.069	2765	76	0.987	2729
0.953	133114	130258	2.2	85	1.037	135024	88	1.035	139789	93	1.057	147732
0.909	51388	53486	-3.9	79	0.988	52817	81	1.025	54155	90	1.111	60172
1.013	7923	7285	8.8	72	0.947	6902	76	1.056	7285	76	1.000	7285
0.974	9394	9208	2.0	76	1.027	9457	80	1.053	9955	85	1.063	10577
0.940	68030	65398	4.0	83	1.051	68709	82	0.988	67881	89	1.085	73676
0.922	33046	28075	17.7	88	1.060	29766	87	0.989	29428	91	1.046	30781
0.940	18913	18381	2.9	79	1.013	18617	86	1.089	20266	95	1.105	22387
0.956	27629	26240	5.3	87	1.000	26240	89	1.023	26843	90	1.011	27145
0.938	26253	24693	6.3	77	1.027	25351	89	1.156	29302	84	0.944	27656
0.874	8511	9151	-7.0	92	1.108	10143	89	0.967	9813	94	1.056	10364
0.988	34591	30603	13.0	85	1.000	30603	86	1.012	30963	87	1.012	31323
0.913	37291	34966	6.6	93	1.107	38712	90	0.968	37464	95	1.056	39545
0.929	53432	51987	2.8	82	1.038	53961	86	1.049	56593	92	1.070	60542
1.024	34400	30507	12.8	85	0.988	30152	82	0.965	29088	86	1.049	30507
0.824	14387	15145	-5.0	83	1.107	16760	89	1.072	17972	84	0.944	16962
0.925	32583	33694	-3.3	86	1.000	33694	90	1.047	35261	93	1.033	36437
0.899	5896	6045	-2.5	68	0.958	5790	73	1.074	6215	81	1.110	6896
1.000	10773	9281	16.1	86	1.012	9390	89	1.035	9718	89	1.000	9718
1.000	13077	14149	-7.6	77	0.975	13791	84	1.091	15045	82	0.976	14686
0.921	8537	8177	4.4	80	0.976	7978	87	1.088	8676	82	0.943	8177
0.988	54603	50347	8.5	84	1.024	51575	83	0.988	50961	92	1.108	56487
0.939	9525	9031	5.5	77	1.000	9031	79	1.026	9266	84	1.063	9852
1.000	120228	105600	13.9	100	1.000	105600	87	0.870	91872	86	0.989	90816
0.977	60829	57243	6.3	80	0.952	54517	85	1.063	57924	81	0.953	55199
0.987	3973	3843	3.4	71	0.910	3498	78	1.099	3843	80	1.026	3942
0.868	62416	65151	-4.2	87	1.101	71749	90	1.034	74223	93	1.033	76697
0.965	20776	19782	5.0	78	0.940	18590	84	1.077	20020	82	0.976	19544
0.910	21614	20141	7.3	76	1.070	21559	76	1.000	21559	77	1.013	21843
0.935	77260	72403	6.7	93	1.069	77396	93	1.000	77396	100	1.075	83222
0.920	6210	5552	11.9	80	0.988	5483	90	1.125	6169	91	1.011	6237
0.879	25385	27362	-7.2	78	0.975	26678	89	1.141	30440	92	1.034	31466
0.864	4451	4738	-6.1	82	1.171	5550	84	1.024	5686	81	0.964	5483
0.857	33675	36396	-7.5	82	0.976	35529	89	1.085	38562	89	1.000	38562
0.987	125843	116875	7.7	75	0.962	112380	83	1.107	124367	81	0.976	121370
0.958	11502	11602	-0.9	71	1.029	11938	71	1.000	11938	78	1.099	13115
0.974	3967	3710	6.9	84	1.120	4155	83	0.988	4106	84	1.012	4155
0.976	42754	39599	8.0	77	0.939	37184	83	1.078	40082	82	0.988	39599
0.900	35873	36018	-0.4	76	1.056	38019	74	0.974	37019	77	1.041	38519
0.910	11249	11445	-1.7	100	1.099	12577	100	1.000	12577	100	1.000	12577
0.849	30647	32962	-7.0	87	1.101	36300	85	0.977	35465	91	1.071	37969
0.914	2789	2857	-2.4	67	1.047	2991	69	1.030	3080	66	0.957	2946
